# T12. VITAMIN D STATUS AND PSYCHOTIC DISORDER: ASSOCIATIONS WITH CLINICAL VARIABLES AND RISK FACTORS

**DOI:** 10.1093/schbul/sby016.288

**Published:** 2018-04-01

**Authors:** Christine van der Leeuw, Lot de Witte, Claude van der Ley, Richard Bruggeman, Jim van Os, Machteld Marcelis

**Affiliations:** 1 Maastricht University Medical Center; 2 Icahn School of Medicine, University Medical Center Utrecht, Utrecht University; 3 University of Groningen, University Medical Center Groningen; 4 University Medical Center Utrecht, Utrecht University, Maastricht University Medical Center, Institute of Psychiatry, King’s College London; 5 Maastricht University Medical Center, Institute for Mental Health Care Eindhoven (GGzE)

## Abstract

**Background:**

The association between schizophrenia and decreased vitamin D levels is well documented. Low maternal and postnatal vitamin D levels suggest a possible etiological mechanism. Vitamin D deficiency in patients diagnosed with schizophrenia is presumably (also) the result of disease-related factors.

Furthermore, certain demographic risk factors such as urbanicity may be associated with vitamin D.

**Methods:**

In a large study population of 347 patients with psychotic disorder and 282 controls, associations between vitamin D levels in blood and clinical variables and risk factors were investigated.

Regression analyses were conducted correcting for gender, age, ethnicity, body mass index (BMI), smoking and sampling season. Group × symptomatology and group × urbanicity interactions were investigated. Both current urbanicity and urbanicity at birth were assessed.

**Results:**

Vitamin D concentrations were significantly lower in patients (B= -8.05; 95% confidence interval (CI) -13.68 to -2.42; p=0.005). There were (trend) significant interactions between group and vitamin D for symptomatology (positive symptoms: χ2=2.81 and p=0.094; negative symptoms: χ2=5.63 and p=0.018). A small but significant effect was detected: higher vitamin D concentration was associated with lower positive (B= -0.02; 95% CI -0.04 to 0.00; p=0.049) and negative symptom levels (B= -0.03; 95% CI -0.05 to -0.01; p=0.008) in patients. The group × current urbanicity interaction was not significant. However, the group × urbanicity at birth was significant when corrected for current urbanicity (χ2=11.26 and p=0.001).

**Discussion:**

Vitamin D levels in patients with psychotic disorder were lower than in controls, with an interaction between group and urbanicity at birth. In the patient group, symptom levels were lower when vitamin D concentration was higher.

